# Perception of the Importance of Inherited Genetics and Lifestyle on the Development of Chronic Diseases: A UK Cross‐Sectional Survey

**DOI:** 10.1002/hsr2.70324

**Published:** 2025-01-05

**Authors:** Aamir Hussain, Lourdes Santos‐Merx, Mariasole Da Boit

**Affiliations:** ^1^ Faculty of Health and Life Sciences, Leicester School of Allied Health Sciences De Montfort University, The Gateway Leicester UK; ^2^ Department of Health, School of Health, Sport and Food University College Birmingham Birmingham UK

**Keywords:** chronic diseases, genetics, lifestyle, perception

## Abstract

**Background and Aims:**

There is emerging evidence that genes, lifestyles and environment play a prominent role in the development of non‐communicable diseases. Currently, there is not information on people's perception of inherited genetics vs. lifestyle on disease development. Knowing people's belief on disease etiology will better inform public health strategies for the adoption of a healthy lifestyle.

**Methods:**

Individuals aged 18 years and over were recruited in 2019 among the UK population and 363 participants completed an anonymous questionnaire. The main outcomes assessed were perception of importance of inherited genes and lifestyle on the development of diseases. *T*‐tests and *χ* ^2^ were used.

**Results:**

The score for the importance of inherited genes in disease development was significantly lower than the lifestyle score (7.5 ± 1.7 vs. 8.5 ± 1, *p* <0.001). Moreover, the majority of the respondents chose lifestyle over inherited genes as most important factor for cancer, cardiovascular disease and Type 2 Diabetes development (50.8% vs. 29.6%, 79% vs. 11.9%, 85.7% vs. 9.6%; respectively), while 60.6% of respondents selected inherited genes for rheumatoid arthritis (*p* < 0.05). Knowledge about genes or a healthy lifestyle did not influence the selection of lifestyle as the most important factor (*p* > 0.05).

**Conclusion:**

Overall, participants were well aware of the importance of lifestyle as a determinant risk factor for chronic disease development. This is an important step toward adopting a healthy lifestyle.

## 1 Introduction

Non‐communicable diseases (NCDs) are a tremendous burden on the NHS. In 2019 it was estimated that 88.8% of deaths in England were attributable to NCDs [1]. It is established that genes, lifestyles, and environment play a prominent role in the incidence and development of NCDs [2, 3] While an individual's genetic pool does not change overtime, modifiable lifestyle factors can greatly influence gene expression and have an impact on the incidence and development of NCDs [4]. For instance, an estimated 30%–50% of all cancers could be prevented and the risks of cardiovascular disease (CVD) and rheumatoid arthritis decreased, by adopting a healthy lifestyle [3, 5, 6, 7, 8].

For this reason, in the UK there have been health promotion campaigns to encourage a healthy lifestyle. For examples, in 2009 Public Health England launched the “Change4Life” program to tackle obesity, and subsequently in 2014 the “Five Year Forward” published by NHS England focused on prevention to better support healthier behaviors [9, 10]. Probably due to this and other public health promotion campaigns, the British general population seem to be familiar with the concept of lifestyle as a determinant for NCDs [11]. While the awareness of the role of lifestyle might come from public health campaigns, for genetics it might be the result of information shared, not only in educational settings, but also by increased reporting of genetic research in the media and participation of the general public in personal genomic research and services [11, 12, 13].

Despite public awareness that both lifestyle and genetics are contributing factors for NCDs, there is not currently information on people's perception of inherited genetics on disease risk. With the increasing popularity and availability of genetic testing, and healthcare providers encouraged to share more knowledge of inherited genes, it is imperative to investigate whether knowledge of inherited genetics might influence people's beliefs on disease risk factors [14, 15]. This is important to evaluate since personal beliefs of disease etiology are linked to perceived abilities of a specific behavior to be effective in reducing disease risk [11, 16]. As such awareness of genetic risk of disease might later influence the adoption of health‐related behaviors [17]. For the same reason, exploring whether perceived knowledge of genes/inherited genes and self‐reported healthy lifestyle can interfere with the perception of lifestyle as determinant factor for disease risk is paramount, as this might affect lifestyle choices and unfavorably impact public health. Moreover, from a public health perspective it would be valuable to know the main channels used by the public to retrieve information on inherited genes and genetics to be able to covey any valuable information in an efficient manner.

Therefore, the main aim of this study was to evaluate the perceived importance of inherited genes and lifestyle on development of chronic diseases. Moreover, we wanted to assess whether the level of perceived knowledge of genetics, and how people consider their own lifestyle to be can interfere with the choice of lifestyle as risk factor; and the common channels used to retrieve information on genetics. We hypothesize that lifestyle will be considered the most important factor for the development of chronic diseases, and the perception of a personal healthy lifestyle will positively influence the choice of lifestyle as determinant factor for diseases, while knowledge of unfavorable inherited genes will make people perceived lifestyle changes as less effective and will likely negatively influence it. Additionally, we hypothesize that education and healthcare providers will be the main sources of genetic information.

## 2 Methods

### 2.1 Study Design and Study Population

A cross‐sectional survey targeting the UK population was carried out in 2019 via a self‐reported questionnaire. Individuals aged 18 years and over, without any pre‐existing conditions related to cancer, T2D, RA, and CVD were able to take part in the study. A power a priori calculation (Qualtrics) with 95% confidence level and 6% of margin error, based on the UK population of 68 million people indicated that the sample should be a minimum of 267.

Questionnaires were administered in two forms: electronically using Google form and paper copies. Informed written consent was obtained before the commencement of the study.

Participants were recruited by providing a study leaflet on the social media platforms Facebook and Instagram, and to friends, colleagues and during outreach events. A QR code on the leaflet was used to give access to participant information, aim of the study, and electronic consent. Once the consent was given participants could access the online questionnaire. For those volunteers preferring a paper version of the questionnaire, a hard copy of the participant information sheet and consent form were provided before completing the questionnaire. The study was approved by De Monfort University ethics committee (ref. 3229).

### 2.2 Questionnaire Design

The questionnaire was anonymous, and questions were grouped into 4 sections: section Background and Aims, 1 covered individual characteristics; section Methods, 2 assessed the perception of the importance of inherited genes and lifestyle on disease formation; section Results, 3 assessed the perception of the importance of inherited genes and lifestyle on NCDs disease development, and probed the perception of knowledge regarding genes and inherited genetic and where people sourced this information; section Conclusion, 4 included a question on perception of personal healthy lifestyle.

Specifically, questions in section Methods, 2 were: *“*How important do you feel inherited genes (genetic information passed from your parents to you) are for the formation of diseases?”; and “How important do you feel lifestyle (e.g. diet, exercise and stress) is for the formation of diseases?”. A 10‐point Likert scale was used for both questions, with the extremes being “Not at all” representing 0 and “Very” representing 10.

Questions in section Results, 3 were: “Which factor do you think is more important for the development of cancer?”; “Which factor do you think is more important for the development of type 2 diabetes?”; “Which factor do you think is more important for the development of cardiovascular disease?” and “Which factor do you think is more important for the development of rheumatoid arthritis?”. An answer with multiple choice was provided for all these questions, with the choices being: “Inherited genes”, “Lifestyle” and “Not sure”, with only one possible choice. The subsequent questions were: “How much do you know about genes in general?”, and “How much do you know about your own set of genes. An answer with multiple choice was provided for all these questions, with the choices being: “Nothing at all”, “Some information” or “Quite a lot”, with only one possible choice. The final questions for this section were: “Where did you obtain/learn the information about genes?” and “Where did you obtain/learn the information about your own set of genes?” both with a multiple‐choice answer including: “N/A”, “NHS General Practitioner (GP)”, “Private healthcare practice”, “School (primary, secondary or College)”, “University”, “Family and friends”, “Media (TV, radio, internet)”, “Social‐media (Facebook, Twitter, Instagram, Snapchat, LinkedIn)”, or “Other”; more than one selection was possible.

Question in section Conclusion, 4 was: “How do you consider your lifestyle”. An answer with multiple choice was provided with the choices being: “Very unhealthy”, “Unhealthy”, “Healthy” and “Very healthy”, with only one possible choice.

### 2.3 Statistical Analysis

The collected data were analyzed using SPSS (version 28.0 software). Descriptive data and *χ* ^2^ test with Bonferroni post hoc test were used to assess related factors associated with knowledge and perception. The factors, divided into sub‐groups and with choices selected by only few respondents gathered together, were education (GCSES/A level, undergraduate, postgraduate (Masters and PhD), other), age (below 29, 30–39, over 40), ethnicity (White, Asian, other [Black, Arab, Mixed]), sex (male, female), occupation (worker [manual, nonmanual, professional], student, unemployed, other) and degree (Health, other [Business and Law; Art, Design and Humanities; Computing, Engineering and Media]). To analyze the impact of these factors on the Likert scale scorings a Kruskal–Wallis and Mann–Whitney *U* tests were used, while a dependent *t*‐test (two‐tailed) was used to assess the difference between Likert scale scorings. A point‐biserial correlation (two‐tailed) was used to assess the strength of association between the perception of their own lifestyle (healthy/unhealthy) and genetics knowledge (genes/inherited genes) vs. perception of importance of inherited genes and lifestyle in the formation of diseases. Answers of the multiple‐choice questions on the source of information were grouped as: health (NHS General Practitioner, Private healthcare practice), education (school, university), family and friends, media (media and social media), and other; while for the personal healthy lifestyle question were grouped as: healthy (very healthy and healthy) and unhealthy (Very unhealthy, unhealthy), before undergoing statistical analysis. Statistical significance was accepted at *p* ≤ 0.05, and all the analyses conducted were pre‐specified.

## 3 Results

For this study self‐reported responses from 363 healthy individuals were considered for analysis. Regarding participants' characteristics some participants did not respond to the question about gender (2), age (17) and residency (3). However, of those who responded, they were mainly from England (80.3%), female (61.2%); aged below 30 (38.7%) or 40 and above (35.3%); White (55.7%) or Asian (30%) origin; working (68.2%) or studying (22.7%); with education level at postgraduate (52.6%) or undergraduate (34.2%); and with a degree in Health (61.6%) (Table 1).

**Table 1:** Respondents' characteristics.

Variables	Total *N* (%)
Gender	
Female	221 (61.2)
Male	140 (38.8)
Age	
≤ 29	134 (38.7)
30–39	90 (26)
≥ 40	122 (35.3)
Ethnicity	
White	202 (55.7)
Asian	109 (30)
Others	52 (14.3)
Occupation	
Worker	247 (68.2)
Student	82 (22.7)
Unemployed	12 (3.3)
Others	21 (5.8)
Education	
GCSE/A levels	37 (10.2)
Undergraduates	124 (34.2)
Postgraduates	191 (52.6)
Others	11 (3)
Degree	
Health	188 (61.6)
Others	117 (38.4)
Residency	
England	289 (80.3)
Wales	35 (9.7)
Scotland	6 (1.7)
Northen Ireland	30 (8.3)

### 3.1 Perception of Importance of Genetic vs. Lifestyle on Chronic Diseases

The Likert scale score for the perception of importance of inherited genes in the formation of diseases was 7.5 ± 1.7 (*n* = 363), while the score for the perception of the importance of lifestyle in the formation of diseases was higher at 8.5 ± 1.4 (*n* = 363). The dependent *t*‐test revealed the 2 Likert scores were significantly different *p* < 0.001, while the Kruskal–Wallis and Mann–Whitney *U* tests showed that perceptions toward both inherited genes and lifestyle were not different across the analyzed related factors (*p* > 0.05).

When investigated whether lifestyle or inherited genes is the most important factor in the development of NCDs, the majority of the respondents chose lifestyle for cancer (*n* = 362), CVD (*n* = 362), and T2D (*n* = 363), and this was significantly different (*p* < 0.05) for all conditions (Table 2). Moreover, for T2D *χ* ^2^ analysis revealed a significant association with ethnicity: Asian people considered lifestyle more as contributing factor than other ethnicities *χ* ^2^(4) = 12.08, *p* = 0.017. A different trend was shown for the development of RA where 60.6% of respondents (*n* = 363) selected inherited genes over lifestyle, and this was significantly different (*p* < 0.05) (Table 2). Particularly, there was a significant association with gender *χ* ^2^(2) = 11.64, *p* = 0.03, females considered genes being more relevant than males; with the type of degree *χ* ^2^(2) = 11.99, *p* = 0.02, people with a degree in Health considered genes more important compared to respondents with other degrees; and with ethnicity *χ* ^2^(4) = 10.58, *p* = 0.032, White people gave more importance to genes than other ethnicities. Lastly, the percentage of people who were unsure of the predominant factor involved varied based on the disease: cancer 19.6%, CVD 9.1%, T2D 4.7%, and RA 22.6%.

**Table 2:** Frequency and percentage of the most important risk factor selected for each non‐communicable diseases (NCDs).

NCDs	Tot (%)		
	Lifestyle	Genes	Not sure
Cancer	184 (50.8)*	107 (29.6)*	71 (19.6)*
T2D	331 (85.7)*	35 (9.6)^a^	17 (4.7)
CVD	286 (79)*	43 (11.9)	33 (9.1)
RA	61 (16.8)	220 (60.6)*	82 (22.6)

Abbreviations: CVD, cardiovascular disease; RA, rheumatoid arthritis; T2D, Type 2 Diabetes.

* *p* < 0.001 different from other conditions.

^a^ *p* < 0.05 different from “Not sure”.

### 3.2 Knowledge About Genes and Sources of Information

**Figure 1:**
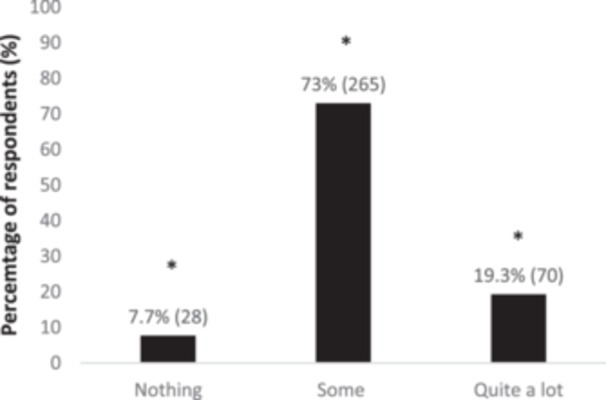
Distribution of answers to the question “How much do you know about genes in general?”. All differences *p* < 0.05; * different from other answers.

**Figure 2:**
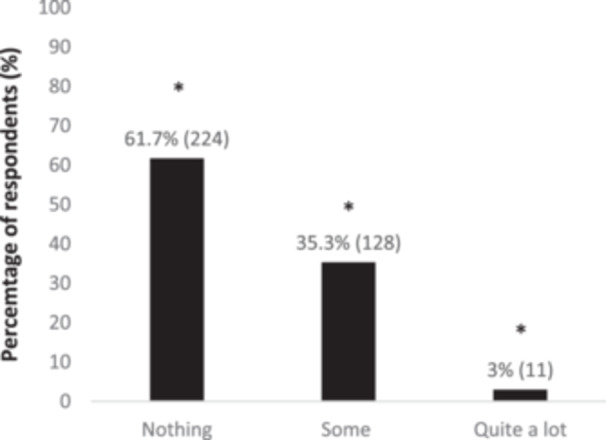
Distribution of answers to the question “How much do you know about your own set of genes?”. All differences *p* < 0.05; * different from other answers.

The majority of the 363 respondents (73%) declared to have some information about genes in general, and this was significantly different (*p* < 0.05) from the other answers, as shown in Figure 1. In particular, there was a significant association with education *χ* ^2^(6) = 20.49, *p* = 0.02, where postgraduates declared to have quite a lot of knowledge more than the other respondents; with the type of degree *χ* ^2^(2) = 28.50, *p* < 0.001, where those with a degree in Health selected some knowledge and quite a lot of knowledge more than people with other degrees; and with occupation *χ* ^2^(6) = 25.91, *p* < 0.001, where students selected quite a lot of knowledge more than the other categories. Of the 363 participants, 61.7% responded to know nothing about their inherited genetic, and this was significantly different (*p* < 0.05), as shown in Figure 2. No differences (*p* > 0.05) were detected after exploring possible correlations with associated factors.

**Figure 3:**
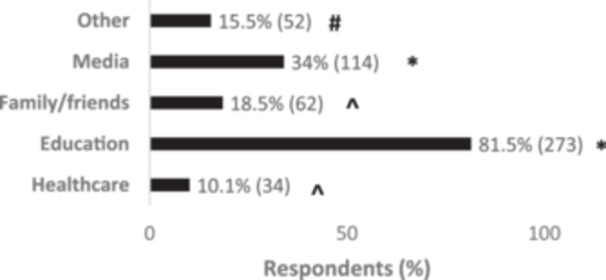
Sources of information about genes in general. All differences *p* < 0.05; * different from all the other options, ^ different from the other options except ‘Other’, # different from media and education.

**Figure 4:**
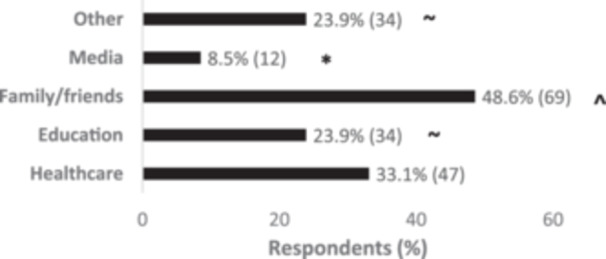
Sources of information about own set of genes. All differences *p* < 0.05; * different from the other options, ^ different from all the other option except ‘Healthcare’, ~ different from media and family.

As source of information, education was selected for genes in general by 81.5% of 335 respondents, and this was significantly different from the other options (*p* < 0.05), (shown in Figure 3). There was a significant association with type of degree *χ* ^2^(1) = 70.73, *p* < 0.001, people with a degree in Health obtain information mainly from education compared to respondents with other degrees; with occupation *χ* ^2^(3) = 16.44, *p* = 0.001, working people gain more information from education compared to the other sub‐groups; and with education *χ* ^2^(3) = 23.48, *p* < 0.001, where postgraduates acquire more information from education than people with other levels of education. On the other hand, over 40 declared to obtain more information from media (46.3%) *χ* ^2^(2) = 14.37, *p* < 0.001, compared to the other age groups, while people with a degree not in Health obtain the majority of information from family and friends *χ* ^2^(1) = 36.22, *p* < 0.001. Whereas the most common source of information selected for inherited genetics was family and friends (48.6% of 142 respondents), and this was significantly different form the other options (*p* < 0.05), except healthcare (33.1%) (shown in Figure 4). Further analysis revealed an association between respondents with a degree in Health and education as source of information (90.3%) *χ* ^2^(1) = 13.21, *p* < 0.001; and between postgraduates and media (58.3%) *χ* ^2^(3) = 8.2, *p* = 0.042.

### 3.3 Influence of Genetic Knowledge and Lifestyle on Disease Risk Factors

Knowledge of genes and inherited genes was not significantly correlated to the choice of disease risk factor (inherited genes vs. lifestyle), for neither the development of a specific NCDs or disease in general (*p* > 0.05).

Of the respondents (*n* = 362) 73.2% declared to have a healthy lifestyle. When exploring whether the perception of participants' own lifestyle was linked to the choice of disease risk factors (inherited genes vs. lifestyle), there was not significant correlation with the factor chosen for neither the development of a specific NCDs or disease in general (*p* > 0.05).

## 4 Discussion

In this study we found that lifestyle was considered to be more important than inherited genes for the development of chronic diseases and in particular cancer, CVD and T2D, but not RA. Furthermore, the majority of respondents reported to have some knowledge about genes but no knowledge of inherited genes, and this, along with self‐reported healthy lifestyle, did not affect the perception of lifestyle as being the most important factor. The most common channels to retrieve information about genetics were education for genes and healthcare and family/friends for inherited genes.

With this study we showed that our sample considered lifestyle to be more important than inherited genes for the development of chronic diseases. These data expand from previous findings in which both lifestyle and genetics were indicated as factors influencing chronic disease risk [11]. This awareness on lifestyle as determinant factor is perhaps the result of public health campaigns aiming to promote a healthier lifestyle to prevent NCDs [18].

In the current study, lifestyle was also selected as the most impactful factor specifically for the development of cancer, CVD and T2D. This is in contrast with previous findings on cancer and CVD, where the UK public showed little understanding of the impact of lifestyle on cancer, and moderate for heart disease, although the profile of the surveyed responded was different from ours [19, 20, 21]. This increased awareness of lifestyle as determinant factor for NCDs might also be the result of the aforementioned health campaigns. On the other hand, our findings on T2D are in agreement with previous research showing participants had adequate awareness of lifestyle choices impacting T2D [22]. Particularly, we found that Asian people considered lifestyle as a contributing factor for the development of T2D in contrast with other ethnicities, perhaps because of a higher incidence of T2D among Asian people, who might be more exposed to information related to prevention of T2D [23]. Differently, RA respondents selected inherited genes. This is perhaps not surprising, as it is still not clear whether some lifestyle factors can have an impact on the incidence and development of RA, with only smoking showing definitive evidence [24]. This uncertainty was also shown by our respondents who selected “not sure” for RA more than for the other NCDs. Additionally, females, people with a degree in Health and White people considered genes being more relevant for arthritis. There is currently no explanation for these differences, and further research should be performed to explore these findings further. Overall, it seems that the public is well aware of the importance of lifestyle in the development of diseases, and this is a fundamental step towards its recognition as effective intervention for disease prevention and, consequently, adoption of a healthy lifestyle [11].

This study shows the majority of the respondents had some knowledge about genes in general, probably coming from education, increased engagement with genetic services, and more reporting of genetic research in the media [11, 12, 13, 25]. Furthermore, students especially postgraduates and those with a degree in Health declared to have more knowledge than the other respondents, as similarly described in a study on the Brunei population [26]. The knowledge of genes in general was obtained mainly from education, and this is not surprising since the main setting where genetics is discussed is education [25]. People with a degree not in Health retrieve genetics information from family and friends, as already reported in the literature for cancer‐related information [27]. Interestingly, respondents over 40 years declared to obtain information from media, however, we would have expected the younger generation to engage more with this source [28]. Education and media should, therefore, be used to convey important information about genetics and health.

Of the participants, 61.7% declared to know nothing about their inherited genetics. This finding was unexpected as there has been an increase in genetic testing and related information [12]. Among those with some knowledge, family and friends were the principal sources of information as previously reported, along with healthcare, maybe due to healthcare providers now encouraged to share genetic information with patients' relatives [14, 27].

From our survey, the level of knowledge about genes in general, about inherited genes and the self‐reported lifestyle were not significantly correlated to the choice of disease risk factor (inherited genes vs lifestyle), for neither the development of a specific NCDs nor disease in general. Consequently, similar to earlier findings, it seems that having some knowledge regarding genetics does not influence the choice of lifestyle as important determinant for health; as well as people might think of healthy lifestyle as effective recommended behavior but might not perceived the threat of a disease enough to adopt that specific behavior [11, 16].

Despite novel findings, our study had some limitations. The main limitation is that the majority of the population probed within this survey had a degree (86.8%), of which 61.6% in Health‐related subjects. According to a recent report 33.8% of people aged 16 years and over had Level 4 or above qualifications in England and Wales [29], therefore, our sample had a high education attainment, particularly health education, and this might have influenced the level of health awareness and genetics knowledge and consequently their views. Additionally, the lifestyle question was based on self‐perception and respondents might have reported a healthier lifestyle, in the same way it is known that people tend to over report vegetable and fruit consumption [30].

Based on these preliminary findings, future studies should include a more diverse sample, closer to the profile of the general public, to confirm whether lifestyle is widely considered the main risk factor for the development of chronic diseases. Moreover, it would be of interest to gather more information on people's attitudes towards the specific lifestyle factors, linked to NCDs to better inform public health policies.

## 5 Conclusion

In our study, the respondents considered lifestyle to be more important than inherited genes for the development of chronic diseases and in particular cancer, CVD and T2D, but not RA. Overall it seems participants were well aware of the importance of lifestyle in the development of diseases. Indeed, having no knowledge of inherited genes, some knowledge about genetics as well as a healthy lifestyle did not seem to interfere with the choice of lifestyle as main risk factor. Believing lifestyle as determinant risk factor for disease development is a fundamental step towards considering it as effective prevention strategy and, eventually, adopting a healthy lifestyle.

## Author Contributions

**Aamir Hussain:** conceptualization, data curation, writing–original draft, writing–review and editing, methodology, formal analysis. **Lourdes Santos‐Merx:** conceptualization, writing–original draft, writing–review and editing, methodology. **Mariasole Da Boit:** conceptualization, methodology, writing–original draft, writing–review and editing, data curation, supervision, formal analysis.

## Ethics Statement

The study was approved by De Monfort University ethics committee on the 12th of February 2019 (ref. 3229).

## Consent

Informed written consent was obtained before the commencement of the study.

## Conflicts of Interest

The authors declare no conflicts of interest.

## Transparency Statement

The lead author Mariasole Da Boit affirms that this manuscript is an honest, accurate, and transparent account of the study being reported; that no important aspects of the study have been omitted; and that any discrepancies from the study as planned (and, if relevant, registered) have been explained.

## Data Availability

The data that support the findings of this study are available from the corresponding author, [M.D.B], upon reasonable request.
